# COVID-19 and the return to head and neck outpatient activity in the United Kingdom: what is the new normal?

**DOI:** 10.1007/s00405-020-06458-x

**Published:** 2020-11-06

**Authors:** Sumrit Bola, Dominic Jaikaransingh, Stuart C Winter

**Affiliations:** 1 Otolaryngology Department, Oxford University NHS Foundation Trust, Headley Way, Oxford, OX3 9DU UK; 2 Nuffield Department of Surgical Sciences, University of Oxford, Oxford, UK

**Keywords:** Outpatient clinic, Otolaryngology, SALT, Air change per hour, Aerosol-generating procedure

## Abstract

**Purpose:**

As surgical specialties now begin the graduated return to elective activity and face-to-face clinics, this paper investigates the current head and neck outpatient practices across the United Kingdom.

**Methods:**

A cross-sectional study comprised of an online 20-item survey was distributed to members of the British Association of Head & Neck Oncologists (BAHNO). The survey was open on a web-based platform and covered topics including safety measures for patients, protective equipment for healthcare staff and protocols for the use of flexible nasendoscopy in the clinic.

**Results:**

The survey was completed by 117 participants covering 66 NHS Trusts across the UK. There was a significant reduction in face-to-face Otolaryngology, Maxillofacial and Speech and Language clinic patients when compared to pre-pandemic numbers (*p* < 0.0001). Risk assessments for flexible nasendoscopy were done for 69% of clinics and 58% had an established protocol. Room downtime after flexible nasendoscopy ranged from 0 to 6 h and there was a significant increase in allocated downtime after a patient had coughed/sneezed (*p* < 0.001). Natural ventilation existed in 36% of clinics and the majority of responders didn’t know the Air Change Per Hour (ACPH) of the clinic room (77%). Where ACPH was known, it often did not match the allocated room downtime.

**Conclusion:**

There is a wide variation in outpatient activity across the United Kingdom, but adaptations are being made to try and maintain staff and patient safety. However, more can still be done by liaising with allied teams to clarify outpatient protocols.

**Electronic supplementary material:**

The online version of this article (10.1007/s00405-020-06458-x) contains supplementary material, which is available to authorized users.

## Introduction

The COVID-19 pandemic caused by the SARS-CoV-2 coronavirus infection has resulted in an unprecedented challenge on healthcare systems worldwide [1, 2]. The reduction in clinical activity and elective surgical work has been due to a number of factors including access to primary care, risk of nosocomial infection and the redeployment of staff [3, 4]. As outpatient services adopt new strategies for the delivery of care, many face-to-face appointments have been cancelled, causing a significant backlog of surgical work.

Early evidence suggested that Head and Neck clinicians, for example, those in specialities such as Otolaryngology and Maxillofacial surgery, were at a particularly high risk of contracting the disease [5]. This was attributed to the elevated viral load found in the nasopharynx [6], close proximity to the oral cavity and the frequency of aerosol-generating procedures [7]. Outpatient management of head and neck cancer extends beyond these two specialities with a broad range of multidisciplinary team members performing at risk procedures such as nasendoscopy, speech valve changes and tracheostomy care.

With no established cure or vaccine for COVID-19 to date, the virus is likely to be a burden for the foreseeable future, and there has been extensive published guidance on working during COVID-19 and the return to elective activity [8, 9]. As the first peak of the pandemic passed across most of Europe [10], surgical departments began a graduated return to selective face-to-face clinics.

### Objective

The aim of this paper was to investigate the current head and neck outpatient practices across the United Kingdom as face-to-face clinics are reinstated. We explore safety measures for patients, protective equipment for healthcare staff and protocols around the use of flexible nasendoscopy (FNE) in the clinic. In particular, we investigate if COVID-19 related safety advice [11] is being implemented appropriately.

## Materials and methods

This cross-sectional study comprised of an online 20-item survey which was distributed via email to members of the British Association of Head & Neck Oncologists (BAHNO). Members include the multi-disciplinary healthcare professionals involved in the study and treatment of head and neck cancer and related conditions. The email invitation was directed at those members who performed flexible nasendoscopy (purposive sampling). Prior to distribution, a pre-test of the survey was sent to senior committee members for question composition feedback. Questions were then modified and transferred to a web-based platform (SurveyMonkey, San Mateo, CA, U.S.A. https://www.surveymonkey.com) where it was completed by a small pilot group (*n* = 5) and these results were not included in the main analysis. The study design was based on established survey reporting guidance [12].

The survey was open from July 1st to July 21st 2020 and email invitations were sent twice during this period. Questions were asked with regards to current and former outpatient practices (Online Appendix A). Topics included:

1. Personal protective equipment used in clinic (PPE)
2. Number of patients booked into a clinic
3. Patient screening prior to the clinic appointment
4. Risk assessment and protocols for flexible nasendoscopy
5. Outpatient room ventilation and room air changes per hour (ACPH)
6. Downtime associated with an Aerosol-Generating Procedure (AGP).

For the purpose of this study, flexible nasendoscopy was not classified as AGP unless the patient had coughed or sneezed during the examination. Downtime of a room was defined as the time when a room was left to ‘rest’ prior to cleaning. In this time period, air particles are allowed to fall to the ground or be replaced by the ventilation system and no staff or patient should re-enter the room.

To prevent the entry of duplicate data from the same hospital, participants were asked to name their place of work. Realising that some NHS Trust may have remote sites for outpatient clinics, entries were only excluded from the study if the place of work and data entry were identical. A paired samples t-test was used to determine if there was a statistically significant difference in the downtime after an AGP and the number of patients booked into a clinic. Data were analysed as means and there was a comparison between current activity and that prior to the pandemic.

## Results

### Demographics

**Fig. 1:**
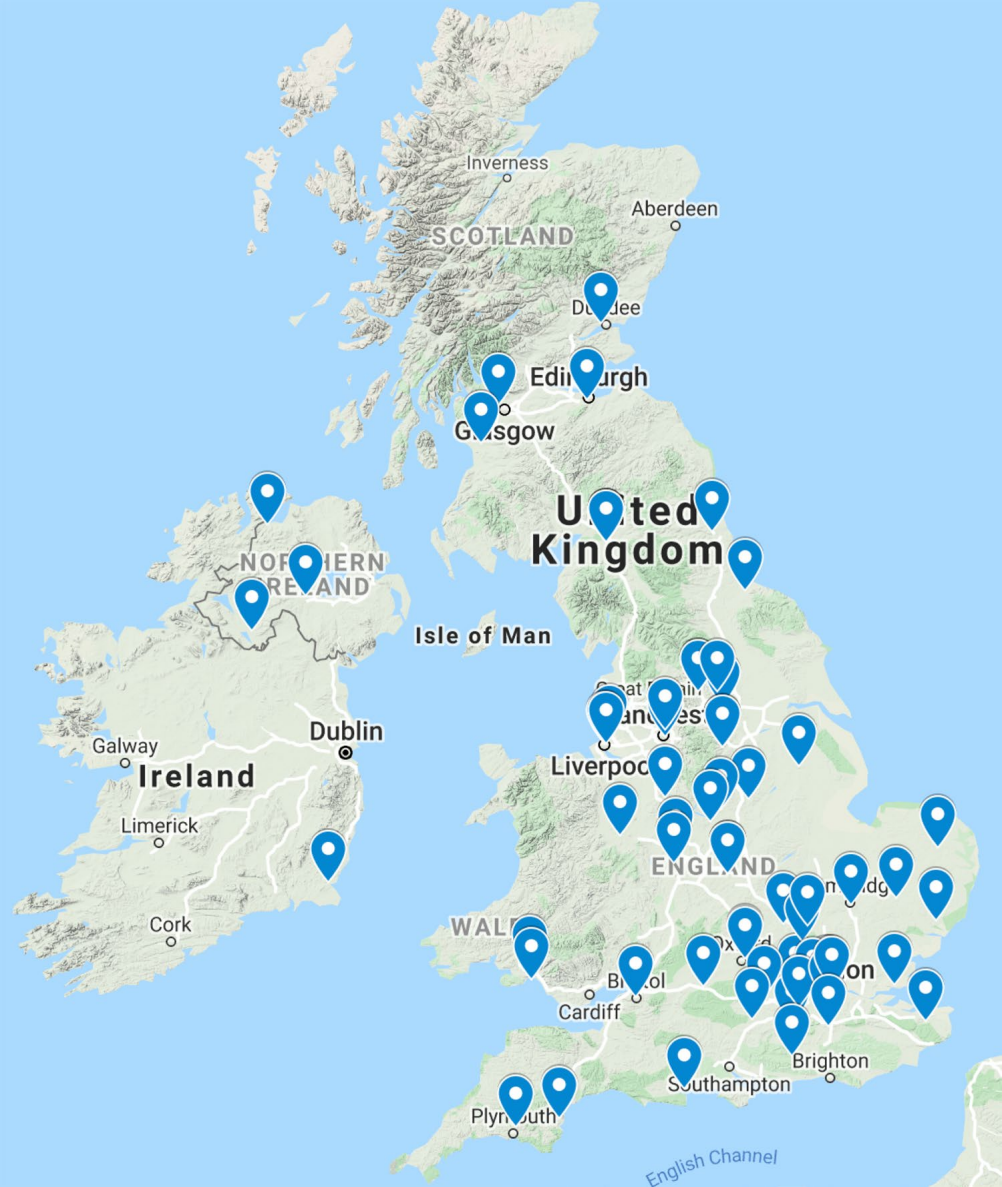
Survey responders across England, Ireland, Scotland and Wales

The survey was sent to 420 members of BAHNO and was completed by 117 (28%) participants. The results captured details of outpatient activity across 66 different NHS Trusts in England, Wales, Scotland and Ireland (Fig. 1). After assessing to see if entries were duplicated, 11 participants were eliminated from the study leaving 106 completed questionnaires for analysis. There was a broad range of responders reflecting the different specialisms who perform nasendoscopy; Otolaryngologists (58%, *n* = 62), Speech and Language Therapists (22%, *n* = 24), Maxillofacial surgeons (16%, *n* = 17) and Oncologists (3%, *n* = 3). It was not known what proportion of the overall membership performed flexible nasendoscopy. The majority of responders were from Tertiary Centres (60%, *n* = 64).

### Patients per clinic

Before the COVID-19 pandemic, the number of patients that otolaryngologists had booked into a 4-h outpatient clinic ranged from 6 to 15 for a single clinic and 14–20 for a joint head and neck cancer (HNC) follow-up clinic, with the majority of clinicians seeing 12 patients (mean = 12.8, standard deviation = 2.5). The post-pandemic figures were between 2 and 14 patients in a 4-h clinic for both single and HNC clinics (mean = 6.5, SD = 2.3), with otolaryngology responders reporting a 50% mean reduction in outpatient appointments (*p* < 0.0001, *t* = 17.28). On average, a new appointment was 1.9 times longer (minutes allocated) than appointments prior to the pandemic. Three otolaryngology responders reported no reduction in booked outpatient clinic numbers; two of these were in district general hospitals and one from a tertiary centre.

Pre-pandemic maxillofacial clinics ranged from 8 to 14 booked patients for a single 4-h clinic and 18–40 patients for a shared HNC follow-up joint clinic (mean = 18, SD = 7.7). Current maxillofacial clinics range from 4 to 10 booked patients for a single clinic and 10–16 for a joint HNC follow up clinic (mean = 7.4, SD = 3.0). Maxillofacial responders reported a significant reduction in patient numbers (*p* < 0.0001, *t* = 5.99) as opposed to the oncology responders who reported that 12–18 patients were booked pre-pandemic versus 6–12 patients post-pandemic; there was no significant difference in post-pandemic activity for oncology.

Speech and Language Therapist reported to having 4–8 patients booked into a specialist clinic (for example voice or dysphagia) and 8–20 into a joint HNC clinic prior to the pandemic (mean = 9.1, SD = 5.5). Post-pandemic numbers were 1–4 and 6–16 patients respectively (mean = 3.9, SD = 3.0). Although one responder reported no change in their numbers for joint HNC clinic, there was an overall significant reduction in booked patients post-pandemic (*p* < 0.0001, *t* = 6.04).

### Patient screening prior to outpatient appointment

Patient screening prior to the outpatient appointment was done with temperature checks, letter advice for self-vetting and symptoms screening by the nurse or receptionist (Table 1). Eighty-seven percent of responders knew the screening methods used and most departments (56%) used two or more screening methods prior to clinical review. One responder reported that COVID-19 swabs were being performed prior to a speech and language clinic for Fibreoptic Endoscopic Evaluation of Swallow (FEES) but swabs were not carried out for the otolaryngology appointments in the same NHS Trust. In two otolaryngology departments, patient symptom screening was done by the clinician prior to entering the clinic room and this was the only method used.

**Table 1:** Screening tools utilised by Otolaryngology, Maxillofacial, Oncology and SALT Outpatient clinics

Screening tool	Number of clinics where screen is implemented
Symptoms screening by nurse or receptionist	58
Patient letter advising not to attend if symptomatic	54
Temperature taken prior to consultation	42
Telephone screening prior to appointment	15
Symptoms screening by a clinician at the consultation	6
COVID swab prior to appointment	2
Hospital entrance questionnaire	1
Don’t know if/how patients are screened	2

*Multiple screening tools implemented for each clinic*

In the majority of clinics, patients were reported to ‘always’ wear a mask during the consultation (62%) or ‘sometimes’ during the consultation (12%). In 25% of departments, mask wearing for patients was not standard practice. Four departments reported the utilisation of a mask with a hole for nasendoscopy. There was no significant difference in the reported frequency of patient mask wearing between Otolaryngology, SALT, Oncology and Maxillofacial responders.

### Flexible nasendoscopy and personal protective equipment

All otolaryngology survey responders were still performing flexible nasendoscopy (FNE) in outpatient clinic whilst 5 non-otolaryngologist reported that FNE was no longer performed due to safety concerns or an unsuitable room. Seventy-three respondents (69%) reported having had a risk assessment for performing FNE in outpatients and of these, 61 (58%) had a protocol for the procedure. Thirty-three responders (31%) reported not having a protocol or not knowing if there was a protocol for performing FNE in an outpatient clinic. Overall, the majority of survey responders found that nasendoscopy took 10 min longer than normal to account for donning/doffing and not having an assistant. This did not include the room cleaning time or room downtime in between patients.

For those still performing FNE in clinic, 94 responders (93%) reported wearing Level 2 personal protective equipment (PPE), two respondents reported wearing Level 3 PPE and the remaining 5 reported that they wore Level 1 PPE. The scope was most often performed in a designated flexible nasendoscopy room that was shared between clinicians (42%) or in a nasendoscopy room allocated to each clinician (30%). When asked how the nasendoscopy room was prepared, 84% of responders had a video screen available and 25% had acquired disposable FNE scopes (Table 2).

**Table 2:** How the allocated room for flexible nasendoscopy is prepared

	Number of respondents
Cleared of unnecessary items	92
Video screen availability	85
Single endoscopist in the room	63
Availability of disposable scopes	25
Surfaces cleaned after the procedure (Clinic staff)	92
Deep clean after the procedure (Cleaners)	3

*Multiple preparations for each room*

### Room ventilation

**Fig. 2:**
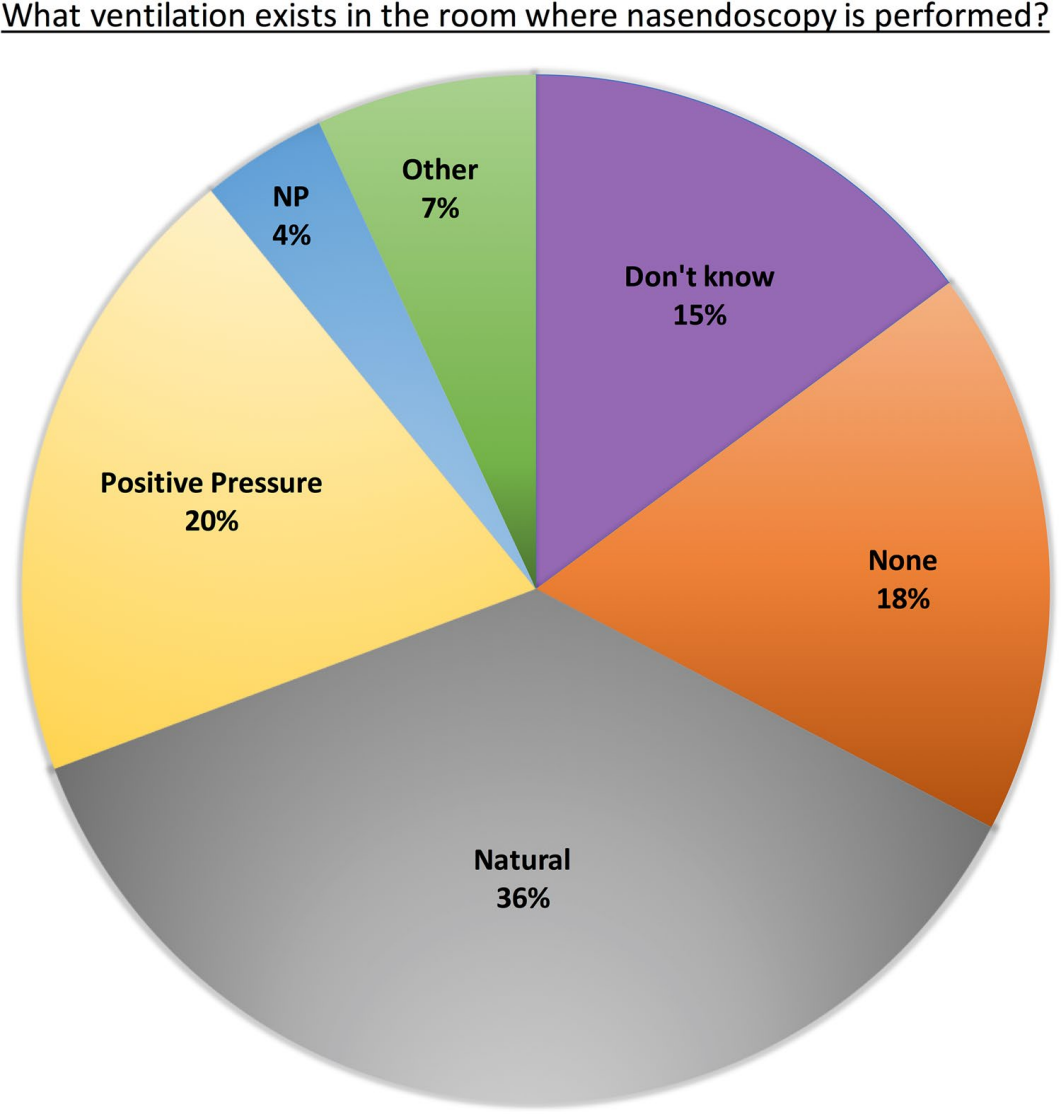
Room ventilation of the allocated flexible nasendoscopy room in the clinic (*NP* Negative pressure)

When asked about the ventilation of the room where flexible nasendoscopy was performed, many responders (*n* = 36) reported natural ventilation only via open windows. Eighteen departments reported that their rooms had no ventilation and 15 responders were unsure what ventilation existed in their nasendoscopy room. Four clinics were described as having negative pressure rooms and 20 responders described having a positive pressure air-conditioned room (Fig. 2).

### Downtime associated with aerosol-generating procedure

**Fig. 3:**
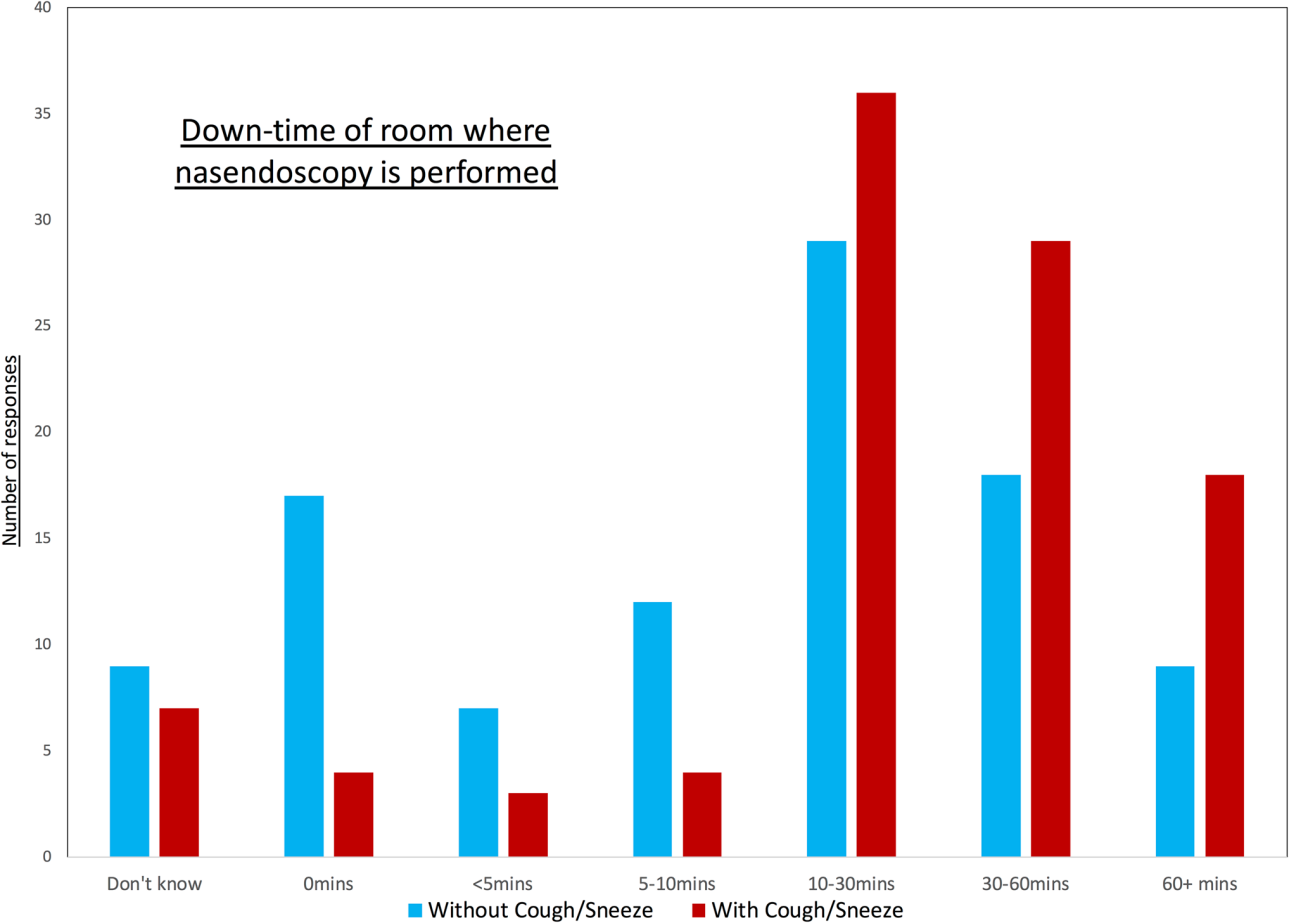
Downtime allocated to flexible nasendoscopy room after an aerosol-generating procedure has taken place. (*Downtime* = *minutes where room is left untouched after patient and clinician have left*)

Survey participants were asked about room downtime when performing flexible nasendoscopy and if there was any difference in downtime if a patient had coughed or sneezed as this was classified as an aerosol-generating event (Fig. 3). From the comments, we found that some rooms were being left as long as 6 h after nasendoscopy. Most departments had a different protocol if an aerosol-generating event had taken place in that the room downtime was longer when the patients had coughed/sneezed compared to when an aerosol-generating event had not taken place (*p* < 0.001).

Twenty-five responders knew the number of air changes per hour (ACPH) of their nasendoscopy room which ranged from 0 to 30 between the clinics (Table 3). In 14 responses, the ACPH did not clear 99% of room air particles in the reported allocated downtime after an AGP [11]. The majority of responders (75%) were unaware of the ACPH of their nasendoscopy room, however, 92% had a policy for room downtime after an AGP.

**Table 3:** Room downtime and Air Changes Per Hour (ACPH) reported by responders

Specialty and number of ACPH of clinic room	Reported downtime after AGP (minutes)	Does this clear 99% of room air particles?	Does this clear 90% of room air particles?
*Otolaryn 0*	10–30	No	No
*Otolaryn 0*	10–30	No	No
*Otolaryn 3*	60 +	Yes	Yes
*Otolaryn 4*	60 +	Yes	Yes
*OMFS 4*	30–60	No	Yes
*OMFS 5*	30–60	Yes	Yes
*Otolaryn 6*	10–30	No	Yes
*Otolaryn 6*	30–60	Yes	Yes
*Otolaryn 6*	60 +	Yes	Yes
*SALT 6*	60 +	Yes	Yes
*SALT 6*	10–30	No	Yes
*OMFS 6*	10–30	No	Yes
*OMFS 6*	30–60	Yes	Yes
*OMFS 6*	5–10	No	No
*OMFS 6*	30–60	Yes	Yes
*Otolaryn 8*	10–30	No	Yes
*Otolaryn 11*	30–60	Yes	Yes
*Otolaryn 12*	10–30	Yes	Yes
*Otolaryn 12*	10–30	Yes	Yes
*Otolaryn 17*	0	No	No
*Otolaryn 18*	10–30	Yes	Yes
*Otolaryn 20*	5–10	No	Yes
*OMFS 22*	10–30	Yes	Yes
*OMFS 25*	5–10	No	Yes
*SALT 30*	10–30	Yes	Yes

*AGP* aerosol-generating procedure, *Otolaryn* Otolaryngology, *SALT* Speech and Language Therapy, *OMFS* Maxillofacial Surgery

## Discussion

These results show a variety of outpatient head and neck clinical activity across the United Kingdom but there were some unifying themes across the different departments. Apart from the Oncologist, all responders reported a significant reduction in outpatient numbers and an increase in the time allocated to each new outpatient appointment. In the comments section, this was attributed to waiting room capacity, availability of audiology, reduced nursing staff and the need to allocate rooms for flexible nasendoscopy. The survey responses did not account for the number of clinics that could run simultaneously, so the estimated reduction in patient numbers is not likely to represent the full extent of the ongoing backlog. It is, therefore, important that strategies are developed to address limited space in outpatient clinics [13].

As social distancing rules continue to be modified in the United Kingdom, it is essential to screen patients for symptoms prior to clinical review. The majority of responders reported at least two COVID-19 screening methods prior to the clinic appointment. Recognising that the virus has a highly infectious but asymptomatic prodrome period, many departments had protocols in place for patients to wear facemasks. There is evidence to support mouth covering reduces droplet production [14] and since the survey closed, it has become compulsory for the public to wear masks whilst indoors [15].

There have been a number of recommendations as to how flexible nasendoscopy (FNE) should be performed safely. Although it is not an aerosol-generating procedure, it involves the manipulation of high viral load nasopharyngeal tissue and may cause an asymptomatic infected patient to cough or sneeze. This results in the aerosolisation of virus particles from the respiratory tract which become airborne and contaminate multiple surfaces in the environment [16]. There were reports of inadequate FNE risk assessments, however, the majority of responders wore Level 2 personal protective equipment for flexible nasendoscopy in the outpatient setting which is in keeping with ENT-UK guidance, Royal College of Speech and Language Therapists guidance and recommendations from outside the United Kingdom [11, 17–19]. Although one SALT department was performing COVID-19 swabs prior to clinic review, there is no guidance recommending this and there is evidence to suggest screening at a time of low virus prevalence can be harmful [20].

In terms of room ventilation and downtime, the protocols for FNE have to be interpreted in the context of each individual hospital; taking into account the physical space and ventilation in which the procedure is taking place. The survival time of SARS-CoV-2 varies considerably by surface type and in aerosol form, viable particles can remain after 3 h [21]. In settings where rooms are re-used after an AGP, this poses a risk not only to clinical staff but ​also ​to subsequent patients entering the room. Although there was less downtime allocated to when a patient had not coughed or sneezed, the allocated downtime and ACPH sometimes did not match. For example, where an ACPH was reported to be 6, we would expect 46 min downtime after an AGP as this removes 99% of the air particles in that room making it safe for the next patient [11]. We instead saw a range of times from 5 min to 6 h after an AGP (patient coughing or sneezing). It was not clear how the downtime was calculated or why any downtime was allocated when a patient had not coughed or sneezed. It was also concerning that even though some rooms had no ventilation, potential AGPs were still taking place.

### Limitations

Electronic surveys are known to have a small response rate. However, the survey email invitation was aimed at those members of BAHNO who performed flexible nasendoscopy and so the actual response rate is likely to be higher than reported. This survey is also limited to head and neck clinics which have taken priority over other elective work and so the figures may not represent the full extent of the surgical backlog amongst the specialties.

## Conclusion

The continuing presence of COVID-19 in the population will prevent the immediate return to pre-pandemic clinical activity [22]. Adaptations are being made across the UK to try and maintain staff and patient safety, but more can still be done by liaising with hospital infectious diseases and the hospital estates team to clarify outpatient protocols.

From this survey, we consider the current standard practice to be:

- Level 2 PPE and video screen availability for nasendoscopy
- Reduced number of patients per face-to-face clinic
- Minimum of two patient-screening methods prior to clinician review
- Patient wears masks during clinical consultation
- Longer downtime for nasendoscopy room when patient has coughed/sneezed.

From this survey, we have found that.

- Many clinicians do not know air changes per hour of their nasendoscopy room
- Most outpatient departments have natural ventilation only
- Level 1 PPE for flexible nasendoscopy is not common
- There will be a considerable backlog of outpatient work.

## Electronic supplementary material

Below is the link to the electronic supplementary material.

Electronic supplementary material 1 (DOCX 13 kb)
