# Supplementary material for: COVID-19 and the return to head and neck outpatient activity in the United Kingdom: what is the new normal?
Source: Eur Arch Otorhinolaryngol. 2020 Nov 6;278(7):2641–8. doi: 10.1007/s00405-020-06458-x (PMC7647194; doi:10.1007/s00405-020-06458-x)
Supplement: Supplementary file 1 — Electronic supplementary material 1 (DOCX 13 kb) [file 405_2020_6458_MOESM1_ESM.docx]

**Appendix A: Copy of unformatted survey questions**

1)What is your role?

2)How best to describe your primary place of work?

3)Are you currently involved in face-to-face clinics?

4a) How many face-to-face appointments were booked into a 4hour clinic pre-COVID?

4b) How many face-to-face appointments are booked into a 4hour clinic post-COVID?

5a)What time was allocated for NEW outpatient face-to-face encounters pre-COVID?

5b)What time is allocated to NEW outpatient face-to-face encounters post-COVID?

6)How are patients screened for COVID-19 prior to outpatient face-to-face appointment? Select from list below.

7)Where is flexible nasendoscopy currently being performed?

8)In the allocated flexible nasendoscopy room, how is the room set up? Select from list below.

9)What is the average time taken to perform flexible nasendoscopy post-COVID (include donning/diffing but not down-time)

10) What is the down-time (minutes that room is not used) after flexible nasendoscopy when a patient has sneezed or coughed?

11) What is the down-time (minutes that room is not used) after flexible nasendoscopy when a patient has NOT sneezed or coughed?

12) Are patient wearing a mask during the clinical consultation?

13) What PPE do you wear when performing flexible nasendoscopy?

14) What ventilation does the outpatient room have where nasendoscopy is performed? (May need to speak to hospital estates)

15) How many air changes occur per hour in the room that nasendoscopy is performed? (Note: General ward 6, Theatre 25, Anaesthetic room 15)

16) Has your hospital done a risk assessment for performing nasendoscopy in outpatients?

17) Please state where your hospital is based (hospital names are requested to avoid duplicate entries but anonymised during review):

18) Please add any further comments below:
